# Supplementary material for: Modeling glioblastoma heterogeneity as a dynamic network of cell states
Source: Mol Syst Biol. 2021 Sep 16;17(9):e10105. doi: 10.15252/msb.202010105 (PMC8444284; doi:10.15252/msb.202010105)
Supplement: Supplementary file 6 — Source Data for Figure 5 [file MSB-17-e10105-s004.zip › Figure5A_sourcedata/GSEA_3071/nosigdb_stateVI.GseaPreranked.1622795169056/GAROFANO_IAVARONE_LIST_OF_NEURAL_PROGENITOR_MARKER_GENES.html]

Details for gene set GAROFANO\_IAVARONE\_LIST\_OF\_NEURAL\_PROGENITOR\_MARKER\_GENES[GSEA]

|  || Dataset | state63071 |
| Phenotype | NoPhenotypeAvailable |
| Upregulated in class | na\_neg |
| GeneSet | GAROFANO\_IAVARONE\_LIST\_OF\_NEURAL\_PROGENITOR\_MARKER\_GENES |
| Enrichment Score (ES) | -0.60034794 |
| Normalized Enrichment Score (NES) | -2.7133667 |
| Nominal p-value | 0.0 |
| FDR q-value | 0.0 |
| FWER p-Value | 0.0 |
Table: GSEA Results Summary

  

Fig 1: Enrichment plot: GAROFANO\_IAVARONE\_LIST\_OF\_NEURAL\_PROGENITOR\_MARKER\_GENES      
 Profile of the Running ES Score & Positions of GeneSet Members on the Rank Ordered List

  

| PROBE | GENE SYMBOL | GENE\_TITLE | RANK IN GENE LIST | RANK METRIC SCORE | RUNNING ES | CORE ENRICHMENT || 1 | WNT7B |  |  | 222 | 0.588 | -0.0483 | No |
| 2 | SFRP1 |  |  | 426 | 0.479 | -0.0939 | No |
| 3 | FOXA2 |  |  | 535 | 0.446 | -0.1137 | No |
| 4 | EFHD2 |  |  | 598 | 0.425 | -0.1212 | No |
| 5 | GLI1 |  |  | 786 | 0.379 | -0.1646 | No |
| 6 | YBX3 |  |  | 855 | 0.368 | -0.1750 | No |
| 7 | VIM |  |  | 897 | 0.361 | -0.1781 | No |
| 8 | SAE1 |  |  | 1340 | 0.299 | -0.2946 | No |
| 9 | RPL22L1 |  |  | 1473 | 0.283 | -0.3249 | No |
| 10 | NUDC |  |  | 1505 | 0.279 | -0.3271 | No |
| 11 | ZIC1 |  |  | 1814 | -0.251 | -0.4073 | No |
| 12 | SLBP |  |  | 1819 | -0.252 | -0.4026 | No |
| 13 | HMGN2 |  |  | 1857 | -0.258 | -0.4069 | No |
| 14 | CKAP2L |  |  | 1884 | -0.262 | -0.4081 | No |
| 15 | MSI1 |  |  | 1982 | -0.275 | -0.4288 | No |
| 16 | DLG1 |  |  | 2065 | -0.290 | -0.4449 | No |
| 17 | NUF2 |  |  | 2076 | -0.291 | -0.4410 | No |
| 18 | DNAJC9 |  |  | 2077 | -0.291 | -0.4342 | No |
| 19 | CKAP2 |  |  | 2109 | -0.299 | -0.4359 | No |
| 20 | LIG1 |  |  | 2290 | -0.331 | -0.4785 | No |
| 21 | PAX6 |  |  | 2291 | -0.331 | -0.4708 | No |
| 22 | PBK |  |  | 2412 | -0.356 | -0.4961 | No |
| 23 | KIF2A |  |  | 2532 | -0.385 | -0.5204 | No |
| 24 | UHRF1 |  |  | 2549 | -0.388 | -0.5159 | No |
| 25 | NDE1 |  |  | 2588 | -0.397 | -0.5172 | No |
| 26 | TCF19 |  |  | 2605 | -0.402 | -0.5124 | No |
| 27 | RAD21 |  |  | 2797 | -0.457 | -0.5551 | No |
| 28 | ARX |  |  | 2960 | -0.520 | -0.5883 | Yes |
| 29 | NES |  |  | 3001 | -0.542 | -0.5868 | Yes |
| 30 | TMPO |  |  | 3022 | -0.552 | -0.5796 | Yes |
| 31 | NASP |  |  | 3049 | -0.563 | -0.5738 | Yes |
| 32 | RRM1 |  |  | 3070 | -0.574 | -0.5660 | Yes |
| 33 | DEK |  |  | 3098 | -0.583 | -0.5600 | Yes |
| 34 | MIS18BP1 |  |  | 3146 | -0.615 | -0.5589 | Yes |
| 35 | NOTCH1 |  |  | 3192 | -0.657 | -0.5562 | Yes |
| 36 | TP53I11 |  |  | 3198 | -0.662 | -0.5422 | Yes |
| 37 | OXCT1 |  |  | 3216 | -0.685 | -0.5310 | Yes |
| 38 | SOX9 |  |  | 3280 | -0.739 | -0.5315 | Yes |
| 39 | EZH2 |  |  | 3282 | -0.741 | -0.5145 | Yes |
| 40 | SALL1 |  |  | 3284 | -0.742 | -0.4976 | Yes |
| 41 | DHFR |  |  | 3303 | -0.765 | -0.4848 | Yes |
| 42 | SOX2 |  |  | 3314 | -0.778 | -0.4696 | Yes |
| 43 | RRM2 |  |  | 3317 | -0.783 | -0.4519 | Yes |
| 44 | DBI |  |  | 3349 | -0.812 | -0.4417 | Yes |
| 45 | HES6 |  |  | 3384 | -0.864 | -0.4311 | Yes |
| 46 | FBXO5 |  |  | 3413 | -0.919 | -0.4176 | Yes |
| 47 | HMGB2 |  |  | 3414 | -0.922 | -0.3962 | Yes |
| 48 | PROM1 |  |  | 3436 | -0.974 | -0.3794 | Yes |
| 49 | DLL3 |  |  | 3460 | -1.030 | -0.3619 | Yes |
| 50 | ESCO2 |  |  | 3479 | -1.062 | -0.3423 | Yes |
| 51 | TCF12 |  |  | 3494 | -1.100 | -0.3206 | Yes |
| 52 | MYO10 |  |  | 3516 | -1.170 | -0.2993 | Yes |
| 53 | NBL1 |  |  | 3535 | -1.240 | -0.2755 | Yes |
| 54 | CLU |  |  | 3601 | -1.801 | -0.2518 | Yes |
| 55 | ASCL1 |  |  | 3604 | -1.822 | -0.2100 | Yes |
| 56 | INSM1 |  |  | 3607 | -1.872 | -0.1671 | Yes |
| 57 | POU3F2 |  |  | 3610 | -1.933 | -0.1227 | Yes |
| 58 | FABP7 |  |  | 3629 | -2.788 | -0.0630 | Yes |
| 59 | GPD1 |  |  | 3631 | -2.806 | 0.0020 | Yes |
Table: GSEA details [plain text format]

  

Fig 2: GAROFANO\_IAVARONE\_LIST\_OF\_NEURAL\_PROGENITOR\_MARKER\_GENES: Random ES distribution      
 Gene set null distribution of ES for **GAROFANO\_IAVARONE\_LIST\_OF\_NEURAL\_PROGENITOR\_MARKER\_GENES**
